# Deep learning approach for automatic segmentation of ulna and radius in dual-energy X-ray imaging

**DOI:** 10.1186/s13244-021-01137-9

**Published:** 2021-12-20

**Authors:** Fan Yang, Xin Weng, Yuehong Miao, Yuhui Wu, Hong Xie, Pinggui Lei

**Affiliations:** 1 School of Biology and Engineering, Guizhou Medical University, Guiyang, Guizhou Province China; 2 Key Laboratory of Biology and Medical Engineering, Guizhou Medical University, Guiyang, Guizhou Province China; 3 Department of Radiology, The Affiliated Hospital of Guizhou Medical University, No. 28, Guiyi Street, Yunyan District, Guiyang, 550004 Guizhou Province China

**Keywords:** Ulna and radius segmentation, Dual-energy X-ray imaging, Deep learning, Residual block

## Abstract

**Background:**

Segmentation of the ulna and radius is a crucial step for the measurement of bone mineral density (BMD) in dual-energy X-ray imaging in patients suspected of having osteoporosis.

**Purpose:**

This work aimed to propose a deep learning approach for the accurate automatic segmentation of the ulna and radius in dual-energy X-ray imaging.

**Methods and materials:**

We developed a deep learning model with residual block (Resblock) for the segmentation of the ulna and radius. Three hundred and sixty subjects were included in the study, and five-fold cross-validation was used to evaluate the performance of the proposed network. The Dice coefficient and Jaccard index were calculated to evaluate the results of segmentation in this study.

**Results:**

The proposed network model had a better segmentation performance than the previous deep learning-based methods with respect to the automatic segmentation of the ulna and radius. The evaluation results suggested that the average Dice coefficients of the ulna and radius were 0.9835 and 0.9874, with average Jaccard indexes of 0.9680 and 0.9751, respectively.

**Conclusion:**

The deep learning-based method developed in this study improved the segmentation performance of the ulna and radius in dual-energy X-ray imaging.

## Keypoints

- Segmentation of the ulna and radius is important for quantifying osteoporosis.
- The present network model had a better segmentation performance than previous methods.
- Development of deep learning-based method has potential application in clinical practice.

## Background

Osteoporosis is a chronic skeletal disease that is caused by bone loss and can harm bone health and increase the risk of fracture [1]. Osteoporosis has a high incidence rate among middle-aged and elderly people, especially women [2, 3]. In addition, osteoporosis is a systemic bone disease that predisposes patients to fracture and is associated with a high disability rate, long treatment cycle, and high cost, which incur a heavy burden on families and society [4]. According to the latest epidemiological survey in China, the prevalence rate of osteoporosis in people over 50 years of age is 19.2% (6% men and 30% women) [5]. Although the incidence rate and disability rate of osteoporosis are high, early diagnosis, improved diet, exercise, and drug treatment can effectively prevent the occurrence of fractures [6–9].

Currently, osteoporosis is often diagnosed by measuring the bone mineral density (BMD) of patients. The methods commonly used for BMD measurement include ultrasound, dual-energy X-ray imaging and quantitative computed tomography (QCT) [10]. Among these, dual-energy X-ray imaging has a higher accuracy than ultrasound and a smaller radiation dose than QCT. The World Health Organization considers the BMD obtained by dual-energy X-ray absorptiometry (DEXA) as the gold-standard for the diagnosis of osteoporosis [11]. Dual-energy X-ray imaging is often applied to the diagnosis of osteoporosis and to predict fracture risk by measuring the BMD of the ulna and radius, lumbar (L1–L4) vertebrae, and femur [12–14]. The segmentation of the bone region, followed by the calculation of the BMD according to the principle of DEXA (the energy attenuation intensity of low-energy and high-energy X-ray passing through human tissue is different) [15, 16], is important steps in BMD measurement.

The accurate segmentation of the ulna and radius, for BMD measurement to diagnose osteoporosis, could be helpful for the early diagnosis and treatment of distal radius fracture, which may be the initial presentation of osteoporosis [17]. For ulna and radius segmentation, many researchers have used image processing technology to solve the segmentation problem. The modified adaptive clustering of the radius and ulna segmentation algorithm was proposed for bone-age assessment [18]. An improved edge-based segmentation technique was used for the segmentation of the radius and ulna bones [19]. The local entropy method was developed for the detection and segmentation of the radius and ulna bones [20]. Furthermore, the dynamic programming algorithm was applied to segment the ulna and radius for single-energy X-ray absorptiometry BMD measurement [21]. However, those methods are easily affected by noise. Therefore, the accuracy and stability of segmentation need to be improved. Recently, a deep learning method has been widely used in medical image analysis [22, 23]. A previous study has reported a deep learning segmentation model for the ulna and radius on DEXA [24]; however, that method did not distinguish between the ulna and radius regions. The U-Net model [25] was used for radius segmentation in wrist X-ray imaging [26]. A fully convolutional network was also applied for distal radius and ulna segmentation from hand X-ray images [27]. Those methods were mainly used for the analysis of single-energy X-ray images; thus, the segmentation performance on dual-energy X-ray images needs to be verified and improved.

As mentioned above, this work presented a deep learning segmentation network for the automatic and accurate segmentation of the ulna and radius using dual-energy X-ray imaging. The designed residual block (Resblock) was combined in U-Net to improve the accuracy of the segmentation procedure.

## Materials and methods

### Materials

**Fig. 1:**
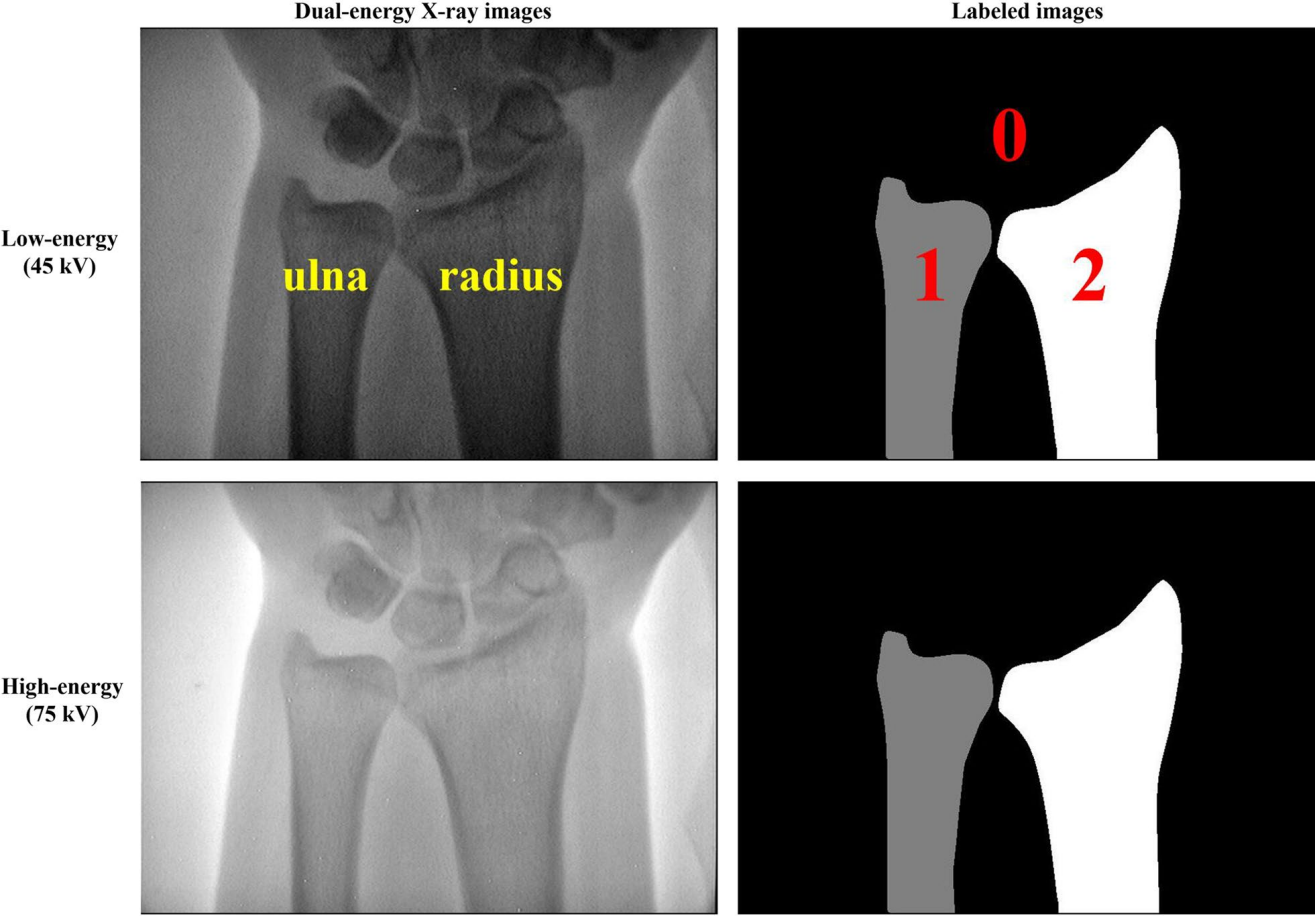
Dual-energy X-ray images and corresponding labeled images. The two images in the first row are the low-energy image and the corresponding labeled image (ground truth). The remaining two images in the second row are the high-energy image and the corresponding labeled image (ground truth). The radius, ulna, and background are labeled as 2, 1, and 0, respectively

The study was approved by the institutional Review Board of Guizhou Medical University. The data obtained from dual-energy X-ray imaging pertained to 360 subjects (171 males; 189 females) aged 36 ± 13 years, with a total of 720 images that were collected using a DEXA-iMAX imaging instrument (Kanrota, Co., Ltd., China). Three hundred subjects with a total of 600 images were used for five-fold cross-validation, and an additional 60 subjects with a total of 120 images were used for independent testing. Each subject yielded two images, i.e., low-energy (45 kV) and high-energy (75 kV) X-ray images (refer to Fig. 1). The ulna and radius regions of each subject were labeled by an experienced radiologist using MIPAV (Medical Image Processing, Analysis, and Visualization) V9.0.0 (https://mipav.cit.nih.gov/). All images had a uniform size of 576 × 768 pixels. The radius, ulna, and background were labeled as 2, 1, and 0, respectively.

### Methods

**Fig. 2:**
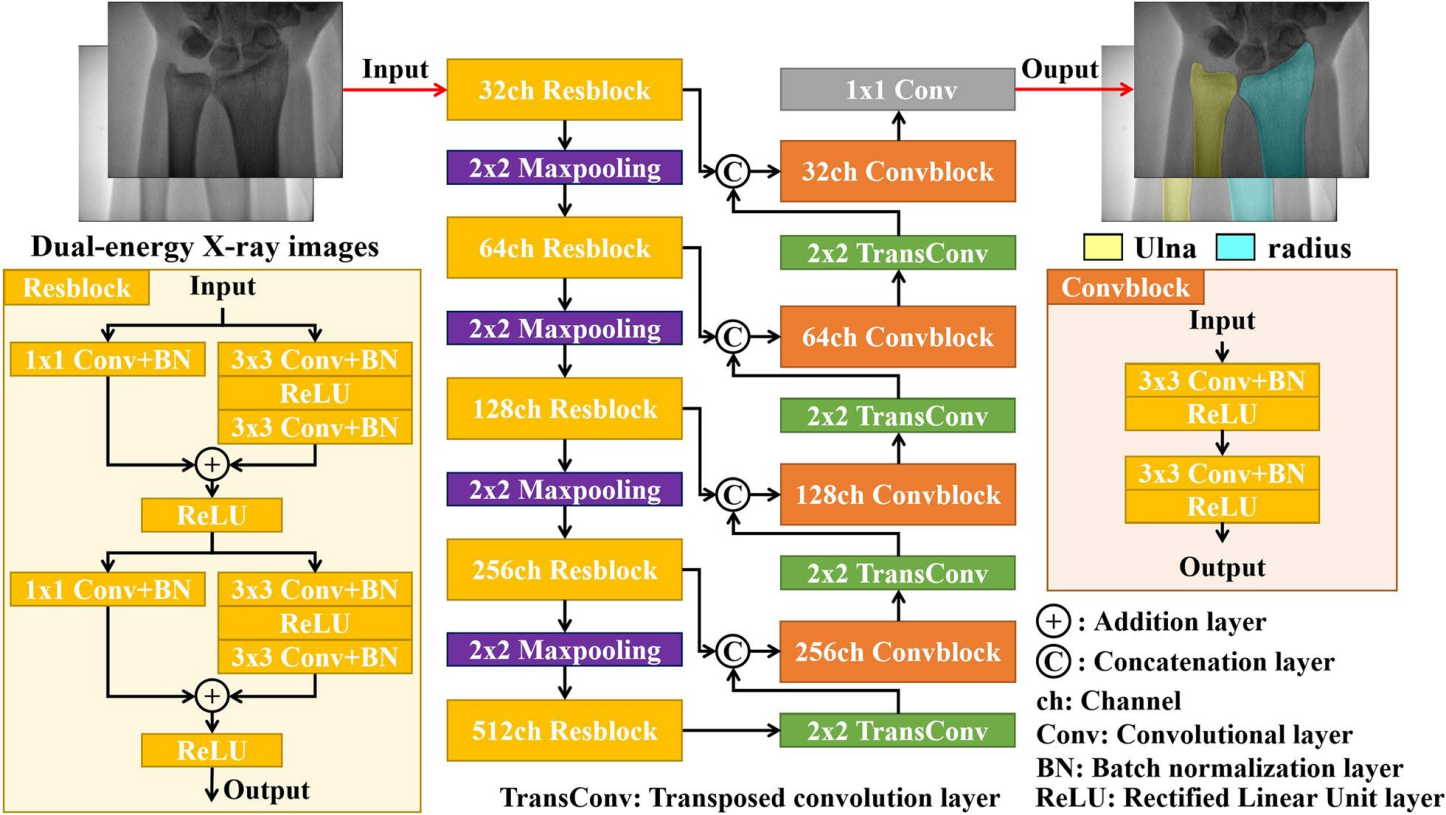
Schematic diagram of the proposed segmentation network for the ulna and radius. The network consists of encoding and decoding stages. The inner structure of the designed Resblock module in the encoding stage is shown in the bottom-left corner of the figure

A schematic diagram of the proposed network is shown in Fig. 2. The network architecture consisted of two stages: encoding and decoding. In the encoding stage, the network included five Resblock modules, four 2 × 2 maxpooling layers, with a size of input the image of 576 × 768 pixels. The Resblock was designed based on ResNet [28] and included four 3 × 3 convolutional layers and two 1 × 1 convolutional layers, which were appended by batch normalization layer (BN) [29] and Rectified Linear Unit layer (ReLU) [30] (see Fig. 2). In the decoding stage, the network included four convolutional blocks (Convblock) and four 2 × 2 transposed convolution layers (TransConv). Each Convblock consisted of two 3 × 3 convolutional layers, BN layers, and ReLU layers, respectively. The number of channels (ch) of each Resblock and Convblock is indicated in Fig. 2. Four skip connections were used to concatenate feature maps along the third channel dimension between the encoding and decoding stages. A 3-channel 1 × 1 convolutional layer was used to map the 32 feature channels to 3 classes (ulna, radius, and background), followed by a softmax layer and loss function layer, to calculate the loss value.

### Loss function

In this work, Generalized Dice Loss [31] was used to compute the total loss of the proposed network. It could alleviate the problem of class imbalance in the image segmentation task. The loss function was as follows:

1 $${\text{Loss}} = 1 - \frac{{2\mathop \sum \nolimits_{c = 1}^{C} w_{{\text{c}}} \mathop \sum \nolimits_{m = 1}^{M} P_{cm} G_{cm} }}{{\mathop \sum \nolimits_{c = 1}^{C} w_{{\text{c}}} \mathop \sum \nolimits_{m = 1}^{M} \left( {P_{cm}^{2} + G_{cm}^{2} } \right)}}$$

2 $$w_{{\text{c}}} = \frac{1}{{\left( {\mathop \sum \nolimits_{m = 1}^{M} G_{cm} } \right)^{2} }},$$

where *P* and *G* denote the predicted image and the corresponding ground truth, respectively; *C* is the number of classes; *M* is the number of elements along the first two dimensions of *P* or *G*; and *w*_c_ is the class weighting factor for each class.

### Implementation details

In the implementation stage, the 300 subjects (600 images) were randomly divided into five folds, three folds for training (180 subjects, 360 images), one fold for validation (60 subjects, 120 images), and one fold for testing (60 subjects, 120 images). Five-fold cross-validation was used to evaluate the performance of the proposed network model. An additional 60 subjects (120 images) were used for independent testing without training. Data augmentation methods were used for all images (360 images) in training sets to prevent overfitting during the training process. The augmentation parameters were as follows: horizontal and vertical translation between − 60 and 60 pixels, horizontal and vertical scaling between 0.9 and 1.1, rotation between − 20° and 20°, and gamma transformation between 0.5 and 1.5.

The network was optimized using the Adam optimizer [32] and model parameters were initialized using He initialization [33]. The network was trained by 500 epochs with an initial learning rate of 0.001, which was reduced by multiplying 0.98 per five epochs, and a mini-batch size of 16. The training set was shuffled in each epoch, and the Dice curve of the mini-batch was used to observe the performance of the training and validation steps. The training process was stopped when no improvement in the Dice score was observed at 500 epochs. The training of 500 epochs for each model required 6⁓7 h for completion. The proposed segmentation network was implemented using the deep learning toolbox of MATLAB 2021a, and our network was trained on a server computer with two Intel® Xeon® Silver 4210 CPUs (2.20 GHz), four NVIDIA RTX 3090 GPUs with 24 GB of memory each, and 128 GB RAM.

### Evaluation metrics

The mean value and standard deviation of the Dice coefficient and Jaccard index were used to evaluate model performance on validation and testing sets. The Dice coefficient was calculated as follows:

3 $${\text{Dice}} = 2\frac{{P_{{\text{c}}} \cap G_{{\text{c}}} }}{{P_{{\text{c}}} + G_{{\text{c}}} }},$$

where *P*_c_ and *G*_c_ denote the predicted image and ground truth of each class (*C* = 1, 2). The Jaccard index for each class was given by:

4 $${\text{Jaccard}} = \frac{{P_{{\text{c}}} \cap G_{{\text{c}}} }}{{P_{{\text{c}}} \cup G_{{\text{c}}} }}.$$

## Results

### Results of segmentation on the validation and testing sets

**Fig. 3:**
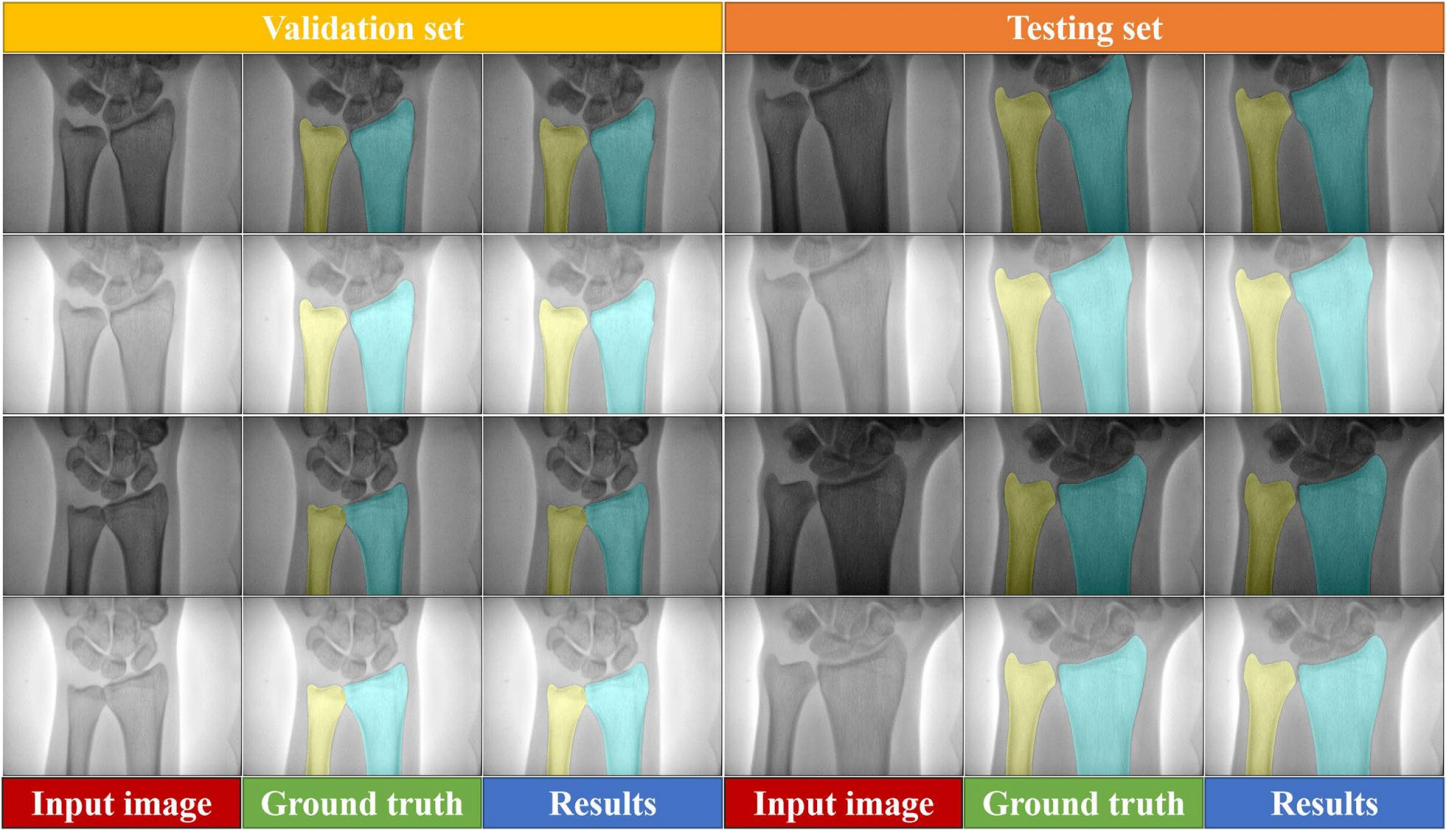
Visualization of the segmentation results for the validation and testing sets. The first and third rows show low-energy X-ray images, and the second and fourth rows show high-energy X-ray images. The first and fourth columns are the input images. The second and fifth columns are the ground truth. The third and sixth columns are the segmentation results obtained using the proposed method. Yellow and cyan denote the ulna and radius, respectively

Five-fold cross-validation was used to evaluate the performance of segmentation on the validation and testing sets. Figure 3 reports the representative segmentation results obtained for the ulna and radius in dual-energy X-ray images using the highest mean Dice score model (yellow and cyan indicate the ulna and radius, respectively). Based on the visual results, the segmentation accuracy of the ulna and radius using the proposed method was comparable to that of manual segmentation, both in low-energy and high-energy X-ray images on the validation and testing sets.

### Comparison with other deep learning-based methods

**Fig. 4:**
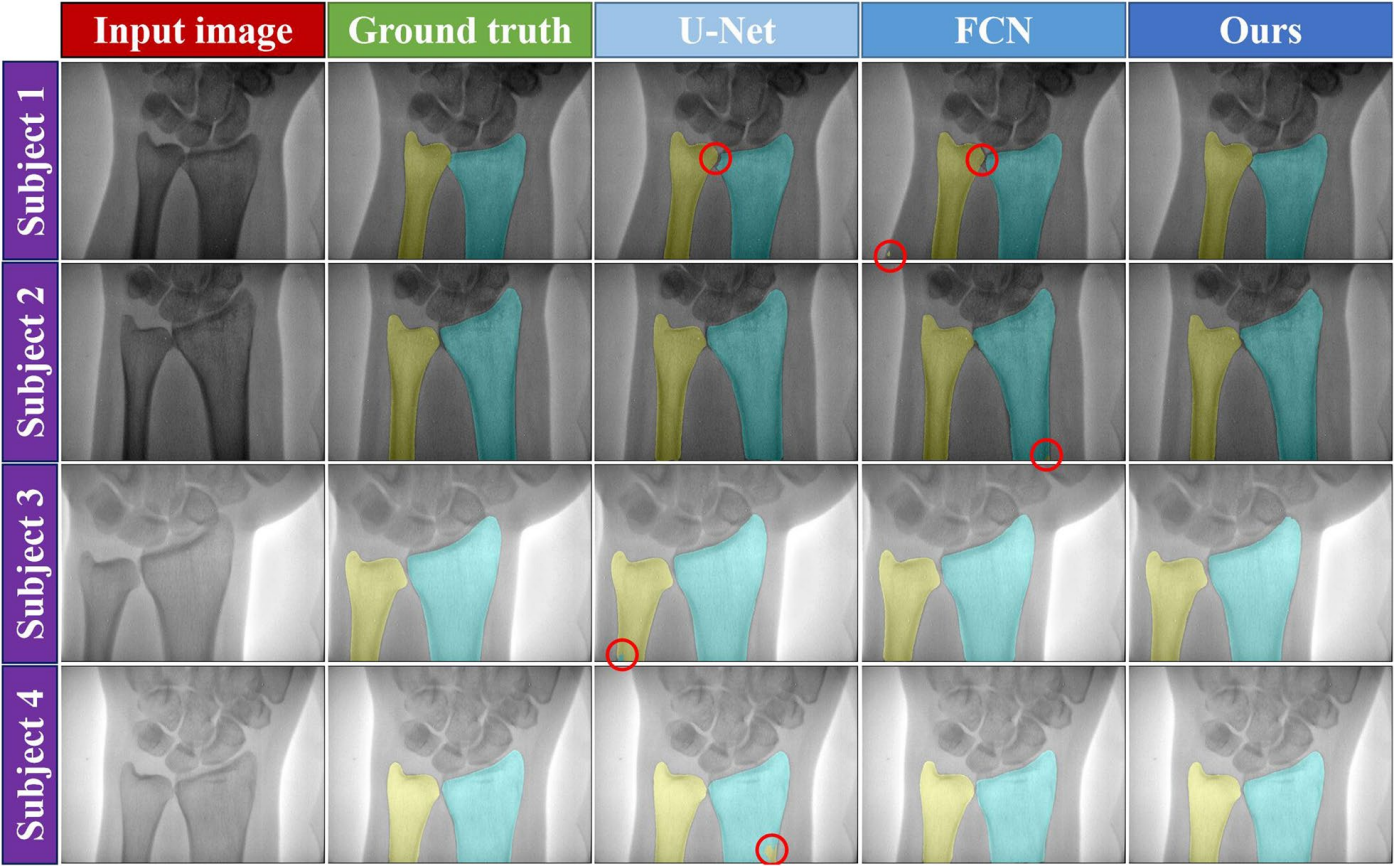
Visual comparison of the ulna and radius segmentation results using different methods on the testing set. Columns from left to right: input image, ground truth, U-Net, FCN, and proposed method. The first and second rows show the low-energy X-ray images, and the third and fourth rows show the high-energy X-ray images. The red circle denotes the region of segmentation error

In references [24] and [27], U-Net and FCN were used to segment the ulna and radius, respectively. We compared those two deep learning-based methods with our network model. All networks were implemented in the same server computer with the same loss function and were trained using the same training options (as detailed in the “Implementation details” subsection). Five-fold cross-validation was also used to evaluate the compared methods. Figure 4 provides a visualization of segmentation results obtained using FCN, U-Net, and our method on the testing set. Based on the visual results, our method had a lower segmentation error than did the U-Net and FCN networks (the segmentation error is marked with a red circle symbol in Fig. 4). Table 1 summarizes the segmentation performance with the evaluation metrics on the validation and testing sets. The Dice coefficient and Jaccard index are the average value of five-fold cross-validation. According to the results, the proposed network model had a better Dice and Jaccard score than that of previous deep learning-based methods for ulna and radius segmentation.

**Table 1:** Quantitative comparison of the validation and testing sets among different methods

Methods	Validation set (Dice)		Testing set (Dice)	
	Ulna	Radius	Ulna	Radius
U-Net	0.9799 ± 0.0228	0.9857 ± 0.0100	0.9804 ± 0.0208	0.9859 ± 0.0093
FCN	0.9786 ± 0.0101	0.9840 ± 0.0061	0.9787 ± 0.0100	0.9841 ± 0.0063
Ours	**0.9838 ± 0.0136**	**0.9874 ± 0.0071**	**0.9835 ± 0.0142**	**0.9874 ± 0.0073**

	Validation set (Jaccard)		Testing set (Jaccard)	
	Ulna	Radius	Ulna	Radius
U-Net	0.9615 ± 0.0400	0.9720 ± 0.0187	0.9624 ± 0.0365	0.9724 ± 0.0176
FCN	0.9582 ± 0.0187	0.9685 ± 0.0115	0.9585 ± 0.0185	0.9688 ± 0.0119
Ours	**0.9684 ± 0.0245**	**0.9752 ± 0.0135**	**0.9680 ± 0.0257**	**0.9751 ± 0.0139**

The results are expressed as the mean ± standard deviation. Bold values indicate the best score obtained for ulna and radius segmentation

### Ablation experimental results

In this section, we conducted an ablation experiment on Resblock to justify the effectiveness of the designed network architecture. Our method redesigned the encoding stage of the U-Net network and replaced it with the Resblock structure. Therefore, we compared the U-Net network (with an initial number of filters of 32 and use of a BN layer after each convolutional layer) with the redesigned network. The same training parameters and loss function (as detailed in the “Implementation details” section) were used without data augmentation. Five-fold cross-validation was also utilized to evaluate the segmentation performance. The experimental results are listed in Table 2. The U-Net with Resblock afforded a higher accuracy than did U-Net without Resblock, according to the Dice coefficient and Jaccard index. This demonstrated the effectiveness of the Resblock architecture on the ulna and radius segmentation performed in this study.

**Table 2:** Quantitative comparison in the presence and absence of Resblock

Methods	Validation set (Dice)		Testing set (Dice)	
	Ulna	Radius	Ulna	Radius
U-Net	0.9753 ± 0.0291	0.9832 ± 0.0126	0.9751 ± 0.0309	0.9828 ± 0.0133
with Resblock	**0.9782 ± 0.0214**	**0.9850 ± 0.0103**	**0.9785 ± 0.0214**	**0.9852 ± 0.0100**

	Validation set (Jaccard)		Testing set (Jaccard)	
	Ulna	Radius	Ulna	Radius
U-Net	0.9534 ± 0.0481	0.9670 ± 0.0233	0.9532 ± 0.0488	0.9665 ± 0.0242
with Resblock	**0.9581 ± 0.0368**	**0.9707 ± 0.0192**	**0.9589 ± 0.0369**	**0.9711 ± 0.0186**

The results are expressed as the mean ± standard deviation. Bold values indicate the best score obtained for ulna and radius segmentation

### Results of segmentation on the independent testing set

To assess the robustness of our method, 60 subjects were used for independent testing without training. Table 3 shows that our algorithm had a better segmentation performance than did other methods, according to the Dice and Jaccard scores.

**Table 3:** Quantitative comparison of the independent testing set among different methods

Methods	Independent testing set (Dice)		Independent testing set (Jaccard)	
	Ulna	Radius	Ulna	Radius
U-Net	0.9767 ± 0.0258	0.9836 ± 0.0114	0.9557 ± 0.0444	0.9681 ± 0.0213
FCN	0.9755 ± 0.0113	0.9824 ± 0.0070	0.9525 ± 0.0212	0.9655 ± 0.0134
Ours	**0.9806 ± 0.0164**	**0.9860 ± 0.0076**	**0.9624 ± 0.0295**	**0.9725 ± 0.0142**

The results are expressed as the mean ± standard deviation. Bold values indicate the best score obtained for ulna and radius segmentation

### Results of the statistical analysis

We used a one-tailed paired *t* test for the evaluation of the results of the validation, testing, and independent testing sets between our method and others. Table 4 lists the results of the statistical analysis, which showed that the proposed method was superior to the previous methods (all *p* values < 0.05).

**Table 4:** Statistical analysis between the proposed method and other methods

Comparison with methods	Validation set (Dice, *p *value)		Testing set (Dice, *p *value)		Independent testing set (Dice, *p *value)	
	Ulna	Radius	Ulna	Radius	Ulna	Radius
U-Net	0.0072	0.0219	0.0110	0.0032	0.0174	0.0070
FCN	0.0004	0.0006	0.0003	0.0002	7.42 × 10^–5^	0.0005

	Validation set (Jaccard, *p *value)		Testing set (Jaccard, *p *value)		Independent testing set (Jaccard, *p *value)	
	Ulna	Radius	Ulna	Radius	Ulna	Radius
U-Net	0.0078	0.0205	0.0102	0.0038	0.0158	0.0057
FCN	0.0003	0.0005	0.0002	0.0002	3.78 × 10^–5^	0.0006

There was a significant difference between the methods when *p* values < 0.05

## Discussion

In this work, we designed a deep learning network with Resblock for accurate ulna and radius segmentation on dual-energy X-ray images. The experimental results based on five-fold cross-validation illustrated that our method had a better segmentation accuracy than did previous deep learning-based methods for ulna and radius segmentation. The proposed method was fully automated without requiring any pre-processing and prior knowledge, and the model could segment about 15 images per second on a NVIDIA RTX 3090 GPU instrument.

The previous methods focused mainly on ulna and radius segmentation on single-energy X-ray images or the segmentation of the ulna and radius as one class in dual-energy X-ray images. Therefore, it is important to propose an accurate segmentation method based on deep learning that segments the ulna and radius as two classes for BMD measurement in dual-energy X-ray imaging. Because the U-Net and FCN networks were previously used in ulna and radius X-ray image segmentation [24, 27], we selected the two networks for comparison with the method proposed here. As the previous data were unavailable, we evaluated these methods based on our dataset. All compared methods used the same training parameters and loss function. Our method designed a Resblock and integrated it into the U-Net network. The Resblock helped the network to alleviate the problem of vanishing gradients and improve the performance of feature extraction. Because of the limited training data, we used a data augmentation strategy during the training, to address the problem of lack of data. The results revealed that the designed network had a lower segmentation error and higher evaluation metrics compared with the U-Net and FCN networks in low-energy and high-energy X-ray images. Furthermore, the current method used smaller datasets and achieved a higher segmentation accuracy compared with the previous methods [24, 27].

BMD is the main method of diagnosing osteoporosis and predicting the risk of fracture [1]. BMD measurement depends on the accuracy of bone segmentation in dual-energy X-ray images. A higher accuracy of the segmentation method may help obtain a more accurate BMD for the diagnosis of osteoporosis using DEXA. Moreover, by collecting more data and combining our segmentation method with regression and classification networks, it will be possible to measure BMD directly and diagnose osteoporosis without segmentation.

The segmentation of the ulna was one of the limitations of this study. The shape of the ulna is more diverse than its radius in different dual-energy X-ray images. The structure of the styloid process of the ulna had a lower segmentation accuracy compared with radius segmentation. The fact that the experimental data of this study were obtained using the same device was another limitation of this study. Uneven exposure also affects the segmentation. The collection of a larger dataset using different dual-energy X-ray imaging devices might help enhance the accuracy and stability of the segmentation process. In addition, the older population who have osteoporosis may have unclear image boundary on ulna and radius compared to younger population in some subjects. It may affect the segmentation accuracy of the proposed method for ulna and radius. However, even if this problem existed, the dice coefficient was only slightly lower than the image with clear boundary. For the deep learning method, collecting similar datasets with unclear boundary or the same age group will help to solve this problem. Further studies could include the application of deep learning for BMD measurement and the diagnosis of osteoporosis.

## Conclusion

This work presented a deep learning segmentation network equipped with Resblock for ulna and radius segmentation in dual-energy X-ray images. The designed Resblock aimed to alleviate the problem of vanishing gradients and help improve the performance of the segmentation of the ulna and radius. We evaluated our network and the recent methods using the same dataset and training parameters. The experimental results showed that presented method segmented the ulna and radius more accurately than did previous methods. We will continue to improve the segmentation accuracy and apply our method to the measurement of BMD and the diagnosis of osteoporosis in future studies.

## Data Availability

The datasets are available from the corresponding author with reasonable request.

## Abbreviations

BMD: Bone mineral density
BN: Batch normalization
DEXA: Dual-energy X-ray absorptiometry
FCN: Fully convolutional network
QCT: Quantitative computed tomography
ReLU: Rectified linear unit
